# 2-(2-Chloro­phen­yl)-3-methyl-5,6-diphenyl-2,3-dihydro­pyrazine

**DOI:** 10.1107/S1600536811036336

**Published:** 2011-09-14

**Authors:** N. Anuradha, S. Chitra, A. Thiruvalluvar, K. Pandiarajan, R. J. Butcher, J. P. Jasinski, J. A. Golen

**Affiliations:** a PG Research Department of Physics, Rajah Serfoji Government College (Autonomous), Thanjavur 613 005, Tamilnadu, India; b Department of Chemistry, K.S.R. College of Engineering, Tiruchengode 637 215, Tamilnadu, India; c Department of Chemistry, Annamalai University, Annamalai Nagar 608 002, Tamilnadu, India; d Department of Chemistry, Howard University, 525 College Street NW, Washington, DC 20059, USA; e Department of Chemistry, Keene State College, 229 Main Street, Keene, NH 03435-2001, USA

## Abstract

In the title mol­ecule, C_23_H_19_ClN_2_, the heterocyclic ring adopts a screw-boat conformation, with all substituents equatorial. The benzene ring at position 2 makes dihedral angles of 77.88 (12) and 76.31 (12)° with the phenyl rings at positions 5 and 6, respectively. The dihedral angle between the phenyl rings at positions 5 and 6 is 70.05 (10)°. The Cl atom is disordered over two positions with occupancy factors of 0.946 (5) and 0.054 (5). In the crystal, C—H⋯π inter­actions are found.

## Related literature

For the biological properties of heterocyclic ring systems having a dihydro­pyrazine nucleus, see: Sondhi *et al.* (2005 ▶). For the use of dihydro­pyrazines, with reference to DNA breakage activity, see: Takechi *et al.* (2011 ▶). For the inhibition of the growth of *Escherichia coli*, see: Takeda *et al.* (2005 ▶). For a closely related crystal structure, see: Anuradha *et al.* (2009 ▶).
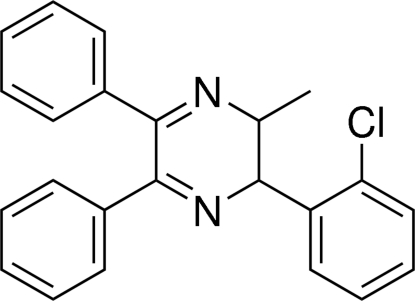


## Experimental

### 

#### Crystal data

C_23_H_19_ClN_2_
*M* *_r_* = 358.85
Monoclinic, 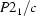

*a* = 10.5675 (8) Å
*b* = 19.7014 (9) Å
*c* = 10.4207 (7) Å
β = 118.479 (9)°
*V* = 1907.0 (3) Å^3^
*Z* = 4
Cu *K*α radiation
μ = 1.82 mm^−1^
*T* = 298 K
0.25 × 0.14 × 0.10 mm

#### Data collection

Oxford Diffraction Xcalibur Eos Gemini diffractometer
Absorption correction: multi-scan (*CrysAlis RED*; Oxford Diffraction, 2010 ▶) *T*_min_ = 0.659, *T*_max_ = 1.000
22812 measured reflections
3831 independent reflections
3092 reflections with *I* > 2σ(*I*)
*R*_int_ = 0.052

#### Refinement

*R*[*F* ^2^ > 2σ(*F* ^2^)] = 0.052
*wR*(*F* ^2^) = 0.150
*S* = 1.04
3831 reflections
240 parameters
2 restraints
H-atom parameters constrained
Δρ_max_ = 0.32 e Å^−3^
Δρ_min_ = −0.28 e Å^−3^

### 

Data collection: *CrysAlis PRO* (Oxford Diffraction, 2010 ▶); cell refinement: *CrysAlis PRO*; data reduction: *CrysAlis RED* (Oxford Diffraction, 2010 ▶); program(s) used to solve structure: *SHELXS97* (Sheldrick, 2008 ▶); program(s) used to refine structure: *SHELXL97* (Sheldrick, 2008 ▶); molecular graphics: *ORTEP-3* (Farrugia, 1997 ▶); software used to prepare material for publication: *PLATON* (Spek, 2009 ▶).

## Supplementary Material

Crystal structure: contains datablock(s) global, I. DOI: 10.1107/S1600536811036336/wn2451sup1.cif

Structure factors: contains datablock(s) I. DOI: 10.1107/S1600536811036336/wn2451Isup2.hkl

Supplementary material file. DOI: 10.1107/S1600536811036336/wn2451Isup3.cml

Additional supplementary materials:  crystallographic information; 3D view; checkCIF report

## Figures and Tables

**Table 1:** Hydrogen-bond geometry (Å, °) *Cg*2, *Cg*3 and *Cg*4 are the centroids of the C21–C26, C51–C56 and C61–C66 rings, respectively.

*D*—H⋯*A*	*D*—H	H⋯*A*	*D*⋯*A*	*D*—H⋯*A*
C24—H24⋯*Cg*4^i^	0.93	2.80	3.643 (3)	152
C53—H53⋯*Cg*2^ii^	0.93	2.99	3.873 (4)	159
C64—H64⋯*Cg*3^iii^	0.93	2.88	3.729 (2)	153

Symmetry codes: (i) 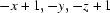; (ii) 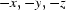; (iii) 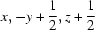.

**Fig. 1.**
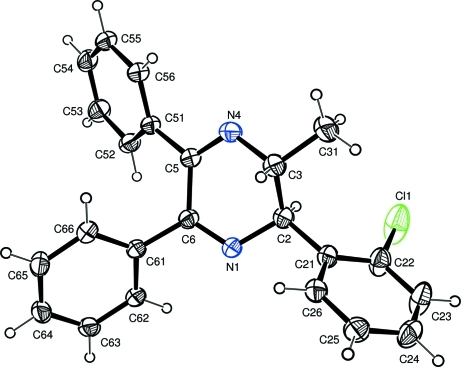
The molecular structure of the title compound, with displacement ellipsoids drawn at the 25% probability level. H atoms are shown as small spheres of arbitrary radius.

## supplementary crystallographic information

### Comment

Heterocyclic ring systems having the dihydropyrazine nucleus have aroused great interest in the past and recent years due to their wide variety of biological properties (Sondhi *et al.*, 2005). Dihydropyrazines are used to break DNA strands and inhibit bacterial growth (Takechi *et al.*, 2011). In addition, these compounds have inhibited the growth of *Escherichia coli* (Takeda *et al.*, 2005). Anuradha *et al.* (2009) have reported the crystal structure of 2-methyl-3,5,6-triphenyl-2,3-dihydropyrazine, in which the heterocyclic ring adopts a screw-boat conformation.

In the title molecule, C_23_H_19_ClN_2_, the heterocyclic ring adopts a screw-boat conformation, with all substituents equatorial. The benzene ring at position 2 makes dihedral angles of 77.88 (12)° and 76.31 (12)° with the phenyl rings at position 5 and 6, respectively. The dihedral angle between the phenyl rings at positions 5 and 6 is 70.05 (10)° (Fig. 1). A C24—H24···π interaction involving the phenyl (C61—C66) ring, a C53—H53···π interaction involving the benzene (C21—C26) ring and a C64—H64···π interaction involving the phenyl (C51—C56) ring are also found in the crystal structure (Table 1). The Cl atom is disordered over two positions. Its occupancy ratio refined to 0.946 (5):0.054 (5).

### Experimental

To a homogeneous solution of benzil (1.05 g, 0.005 mol) and 1-methyl-2-(2'-chlorophenyl)-ethanediamine dihydrochloride (1.29 g, 0.005 mol) in ethanol (20 ml), sodium acetate trihydrate (2.04 g, 0.015 mol) was added. The precipitated sodium chloride was filtered off and the filtrate was refluxed for 2 h. On completion of the reaction, as indicated by TLC, the reaction mixture was poured into crushed ice and the resulting solid was filtered and purified by column chromatography on silica gel. Elution with benzene-petroleum ether (3:2 *v*/*v*) at 333–353 K gave the pure product (1.68 g) in 76% yield. Crystals suitable for X-ray diffraction studies were obtained by recrystallization of the pure product from ethyl acetate.

### Refinement

H atoms were positioned geometrically and allowed to ride on their parent atoms, with Csp^2^—H = 0.93 Å, C(methine)—H = 0.98 Å and C(methyl)—H = 0.96 Å; *U*_iso_(H) = x*U*_eq_(C), where x = 1.5 for methyl H and 1.2 for all other H atoms. The Cl atom is disordered over two positions. Its occupancy ratio refined to 0.946 (5):0.054 (5).

### Crystal data

**Table:**

C_23_H_19_ClN_2_	*F*(000) = 752
*M**_r_* = 358.85	*D*_x_ = 1.250 Mg m^−^^3^
Monoclinic, *P*2_1_/*c*	Melting point: 417 K
Hall symbol: -P 2ybc	Cu *K*α radiation, λ = 1.54184 Å
*a* = 10.5675 (8) Å	Cell parameters from 5083 reflections
*b* = 19.7014 (9) Å	θ = 4.5–73.5°
*c* = 10.4207 (7) Å	µ = 1.82 mm^−^^1^
β = 118.479 (9)°	*T* = 298 K
*V* = 1907.0 (3) Å^3^	Block, pale-yellow
*Z* = 4	0.25 × 0.14 × 0.10 mm

### Data collection

**Table:**

Oxford Diffraction Xcalibur Eos Gemini diffractometer	3831 independent reflections
Radiation source: Enhance (Cu) X-ray Source	3092 reflections with *I* > 2σ(*I*)
graphite	*R*_int_ = 0.052
Detector resolution: 16.1500 pixels mm^-1^	θ_max_ = 73.7°, θ_min_ = 4.5°
ω scans	*h* = −13→12
Absorption correction: multi-scan (*CrysAlis RED*; Oxford Diffraction, 2010)	*k* = −21→24
*T*_min_ = 0.659, *T*_max_ = 1.000	*l* = −12→12
22812 measured reflections	

### Refinement

**Table:**

Refinement on *F*^2^	Primary atom site location: structure-invariant direct methods
Least-squares matrix: full	Secondary atom site location: difference Fourier map
*R*[*F*^2^ > 2σ(*F*^2^)] = 0.052	Hydrogen site location: inferred from neighbouring sites
*wR*(*F*^2^) = 0.150	H-atom parameters constrained
*S* = 1.04	*w* = 1/[σ^2^(*F*_o_^2^) + (0.0681*P*)^2^ + 0.4958*P*] where *P* = (*F*_o_^2^ + 2*F*_c_^2^)/3
3831 reflections	(Δ/σ)_max_ = 0.001
240 parameters	Δρ_max_ = 0.32 e Å^−^^3^
2 restraints	Δρ_min_ = −0.28 e Å^−^^3^

### Special details

**Table:**

Geometry. Bond distances, angles *etc*. have been calculated using the rounded fractional coordinates. All su's are estimated from the variances of the (full) variance-covariance matrix. The cell e.s.d.'s are taken into account in the estimation of distances, angles and torsion angles
Refinement. Refinement of *F*^2^ against ALL reflections. The weighted *R*-factor *wR* and goodness of fit *S* are based on *F*^2^, conventional *R*-factors *R* are based on *F*, with *F* set to zero for negative *F*^2^. The threshold expression of *F*^2^ > 2σ(*F*^2^) is used only for calculating *R*-factors(gt) *etc*. and is not relevant to the choice of reflections for refinement. *R*-factors based on *F*^2^ are statistically about twice as large as those based on *F*, and *R*- factors based on ALL data will be even larger.To allow for a stable and meaningful refinement of the Cl atoms, the C—Cl bonding distances were restrained to be the same (*DFIX* 1.76 0.02 C22 Cl1 C22 Cl2 and EADP Cl1 Cl2).

### Fractional atomic coordinates and isotropic or equivalent isotropic displacement parameters (Å^2^)

**Table:**

	*x*	*y*	*z*	*U*_iso_*/*U*_eq_	Occ. (<1)
Cl1	0.25340 (11)	−0.21842 (8)	0.16414 (14)	0.1246 (4)	0.946 (5)
N1	0.24840 (17)	−0.00156 (8)	0.13885 (16)	0.0488 (5)	
N4	0.2879 (2)	−0.02095 (9)	−0.10830 (18)	0.0627 (6)	
C2	0.3045 (2)	−0.06586 (10)	0.1173 (2)	0.0556 (6)	
C3	0.3806 (3)	−0.05552 (12)	0.0291 (3)	0.0657 (8)	
C5	0.2014 (2)	0.02435 (9)	−0.10666 (19)	0.0479 (5)	
C6	0.20118 (18)	0.04090 (9)	0.03284 (18)	0.0439 (5)	
C21	0.3972 (2)	−0.09884 (10)	0.2638 (2)	0.0527 (6)	
C22	0.3840 (3)	−0.16562 (12)	0.2953 (3)	0.0698 (7)	
C23	0.4712 (3)	−0.19357 (14)	0.4313 (3)	0.0861 (9)	
C24	0.5737 (3)	−0.15478 (14)	0.5392 (3)	0.0767 (8)	
C25	0.5914 (3)	−0.08844 (13)	0.5117 (2)	0.0664 (7)	
C26	0.5037 (2)	−0.06128 (10)	0.3758 (2)	0.0571 (6)	
C31	0.4413 (3)	−0.11844 (14)	−0.0028 (3)	0.0828 (10)	
C51	0.1010 (2)	0.05635 (9)	−0.24852 (19)	0.0474 (5)	
C52	−0.0386 (2)	0.07317 (11)	−0.2826 (2)	0.0562 (6)	
C53	−0.1342 (3)	0.09626 (12)	−0.4219 (2)	0.0664 (7)	
C54	−0.0896 (3)	0.10359 (11)	−0.5258 (2)	0.0682 (8)	
C55	0.0493 (3)	0.08806 (11)	−0.4913 (2)	0.0650 (8)	
C56	0.1443 (2)	0.06438 (10)	−0.3546 (2)	0.0552 (6)	
C61	0.15526 (19)	0.10853 (9)	0.05924 (18)	0.0441 (5)	
C62	0.0827 (2)	0.11347 (9)	0.14084 (19)	0.0478 (5)	
C63	0.0489 (2)	0.17630 (11)	0.1757 (2)	0.0568 (6)	
C64	0.0894 (3)	0.23491 (10)	0.1328 (2)	0.0619 (7)	
C65	0.1610 (3)	0.23089 (10)	0.0524 (2)	0.0638 (7)	
C66	0.1926 (2)	0.16820 (10)	0.0139 (2)	0.0563 (6)	
Cl2	0.249 (2)	−0.1906 (15)	0.131 (2)	0.1246 (4)	0.054 (5)
H2	0.22221	−0.09585	0.06166	0.0668*	
H3	0.46199	−0.02520	0.08554	0.0789*	
H23	0.45990	−0.23883	0.44909	0.1033*	
H24	0.63129	−0.17328	0.63114	0.0921*	
H25	0.66209	−0.06190	0.58423	0.0797*	
H26	0.51645	−0.01613	0.35854	0.0686*	
H31A	0.36507	−0.15035	−0.05490	0.1243*	
H31B	0.51148	−0.13840	0.08724	0.1243*	
H31C	0.48607	−0.10673	−0.06119	0.1243*	
H52	−0.06864	0.06904	−0.21230	0.0674*	
H53	−0.22846	0.10679	−0.44500	0.0797*	
H54	−0.15360	0.11902	−0.61885	0.0818*	
H55	0.07976	0.09357	−0.56100	0.0780*	
H56	0.23810	0.05365	−0.33281	0.0662*	
H62	0.05680	0.07420	0.17213	0.0574*	
H63	−0.00147	0.17897	0.22840	0.0682*	
H64	0.06832	0.27704	0.15826	0.0743*	
H65	0.18848	0.27043	0.02348	0.0765*	
H66	0.23901	0.16601	−0.04263	0.0676*	

### Atomic displacement parameters (Å^2^)

**Table:**

	*U*^11^	*U*^22^	*U*^33^	*U*^12^	*U*^13^	*U*^23^
Cl1	0.1170 (7)	0.0679 (8)	0.1060 (7)	−0.0285 (5)	−0.0141 (5)	0.0118 (5)
N1	0.0561 (9)	0.0475 (8)	0.0414 (7)	0.0091 (7)	0.0221 (7)	0.0047 (6)
N4	0.0780 (12)	0.0660 (11)	0.0473 (9)	0.0211 (9)	0.0324 (9)	0.0053 (8)
C2	0.0608 (11)	0.0550 (11)	0.0456 (10)	0.0144 (9)	0.0209 (9)	0.0056 (8)
C3	0.0808 (14)	0.0633 (13)	0.0593 (12)	0.0228 (11)	0.0384 (11)	0.0090 (9)
C5	0.0557 (10)	0.0474 (9)	0.0414 (9)	0.0020 (8)	0.0239 (8)	0.0012 (7)
C6	0.0445 (8)	0.0467 (9)	0.0393 (8)	0.0013 (7)	0.0189 (7)	0.0023 (7)
C21	0.0556 (10)	0.0504 (10)	0.0464 (10)	0.0127 (8)	0.0198 (8)	0.0053 (8)
C22	0.0627 (12)	0.0561 (12)	0.0651 (13)	0.0016 (10)	0.0098 (10)	0.0082 (10)
C23	0.0831 (17)	0.0629 (14)	0.0829 (17)	0.0048 (12)	0.0158 (14)	0.0281 (13)
C24	0.0715 (14)	0.0817 (16)	0.0546 (12)	0.0177 (12)	0.0120 (11)	0.0200 (11)
C25	0.0633 (12)	0.0718 (14)	0.0496 (11)	0.0085 (10)	0.0151 (10)	−0.0023 (9)
C26	0.0647 (12)	0.0514 (10)	0.0512 (10)	0.0083 (9)	0.0243 (9)	0.0015 (8)
C31	0.0993 (19)	0.0769 (16)	0.0746 (16)	0.0360 (14)	0.0434 (15)	0.0067 (12)
C51	0.0601 (10)	0.0442 (9)	0.0375 (8)	−0.0005 (8)	0.0229 (8)	0.0002 (7)
C52	0.0614 (11)	0.0619 (12)	0.0456 (10)	0.0028 (9)	0.0258 (9)	0.0055 (8)
C53	0.0621 (12)	0.0698 (14)	0.0523 (11)	0.0071 (10)	0.0151 (10)	0.0049 (10)
C54	0.0925 (17)	0.0563 (12)	0.0389 (10)	0.0054 (11)	0.0177 (10)	0.0057 (8)
C55	0.1014 (18)	0.0541 (11)	0.0450 (10)	0.0006 (11)	0.0394 (11)	0.0034 (8)
C56	0.0717 (12)	0.0527 (10)	0.0476 (10)	0.0014 (9)	0.0336 (10)	0.0007 (8)
C61	0.0484 (9)	0.0446 (9)	0.0340 (8)	0.0047 (7)	0.0153 (7)	0.0027 (6)
C62	0.0521 (10)	0.0500 (10)	0.0378 (8)	0.0048 (8)	0.0185 (7)	0.0044 (7)
C63	0.0652 (12)	0.0610 (12)	0.0418 (9)	0.0151 (9)	0.0235 (9)	0.0022 (8)
C64	0.0768 (14)	0.0473 (10)	0.0496 (10)	0.0156 (9)	0.0204 (10)	0.0003 (8)
C65	0.0806 (14)	0.0430 (10)	0.0615 (12)	0.0031 (9)	0.0288 (11)	0.0079 (9)
C66	0.0666 (12)	0.0526 (10)	0.0526 (10)	0.0043 (9)	0.0307 (9)	0.0085 (8)
Cl2	0.1170 (7)	0.0679 (8)	0.1060 (7)	−0.0285 (5)	−0.0141 (5)	0.0118 (5)

### Geometric parameters (Å, °)

**Table:**

Cl1—C22	1.748 (3)	C61—C62	1.394 (3)
Cl2—C22	1.70 (2)	C62—C63	1.384 (3)
N1—C6	1.282 (2)	C63—C64	1.378 (3)
N1—C2	1.461 (3)	C64—C65	1.373 (4)
N4—C3	1.462 (3)	C65—C66	1.388 (3)
N4—C5	1.284 (3)	C2—H2	0.9800
C2—C3	1.496 (4)	C3—H3	0.9800
C2—C21	1.512 (3)	C23—H23	0.9300
C3—C31	1.504 (4)	C24—H24	0.9300
C5—C51	1.488 (3)	C25—H25	0.9300
C5—C6	1.491 (3)	C26—H26	0.9300
C6—C61	1.488 (3)	C31—H31A	0.9600
C21—C26	1.387 (3)	C31—H31B	0.9600
C21—C22	1.379 (3)	C31—H31C	0.9600
C22—C23	1.384 (4)	C52—H52	0.9300
C23—C24	1.364 (4)	C53—H53	0.9300
C24—C25	1.370 (4)	C54—H54	0.9300
C25—C26	1.378 (3)	C55—H55	0.9300
C51—C52	1.383 (3)	C56—H56	0.9300
C51—C56	1.392 (3)	C62—H62	0.9300
C52—C53	1.392 (3)	C63—H63	0.9300
C53—C54	1.379 (4)	C64—H64	0.9300
C54—C55	1.369 (5)	C65—H65	0.9300
C55—C56	1.375 (3)	C66—H66	0.9300
C61—C66	1.392 (3)		
			
C2—N1—C6	116.95 (16)	C61—C66—C65	120.5 (2)
C3—N4—C5	117.38 (19)	N1—C2—H2	108.00
N1—C2—C3	110.66 (17)	C3—C2—H2	108.00
N1—C2—C21	109.42 (15)	C21—C2—H2	108.00
C3—C2—C21	113.6 (2)	N4—C3—H3	107.00
N4—C3—C2	111.1 (2)	C2—C3—H3	107.00
N4—C3—C31	109.0 (2)	C31—C3—H3	107.00
C2—C3—C31	115.8 (2)	C22—C23—H23	120.00
N4—C5—C6	119.82 (16)	C24—C23—H23	120.00
N4—C5—C51	117.16 (17)	C23—C24—H24	120.00
C6—C5—C51	122.98 (18)	C25—C24—H24	120.00
N1—C6—C5	121.10 (17)	C24—C25—H25	120.00
N1—C6—C61	116.89 (16)	C26—C25—H25	120.00
C5—C6—C61	121.90 (15)	C21—C26—H26	119.00
C2—C21—C22	124.1 (2)	C25—C26—H26	119.00
C2—C21—C26	119.70 (18)	C3—C31—H31A	109.00
C22—C21—C26	116.25 (19)	C3—C31—H31B	109.00
Cl1—C22—C21	120.9 (2)	C3—C31—H31C	109.00
Cl1—C22—C23	117.1 (2)	H31A—C31—H31B	109.00
C21—C22—C23	122.0 (2)	H31A—C31—H31C	109.00
Cl2—C22—C21	99.7 (10)	H31B—C31—H31C	109.00
Cl2—C22—C23	138.3 (10)	C51—C52—H52	120.00
C22—C23—C24	120.0 (3)	C53—C52—H52	120.00
C23—C24—C25	119.8 (2)	C52—C53—H53	120.00
C24—C25—C26	119.6 (2)	C54—C53—H53	120.00
C21—C26—C25	122.4 (2)	C53—C54—H54	120.00
C5—C51—C52	121.65 (19)	C55—C54—H54	120.00
C5—C51—C56	119.2 (2)	C54—C55—H55	120.00
C52—C51—C56	118.88 (17)	C56—C55—H55	120.00
C51—C52—C53	120.1 (2)	C51—C56—H56	120.00
C52—C53—C54	120.2 (3)	C55—C56—H56	120.00
C53—C54—C55	119.7 (2)	C61—C62—H62	120.00
C54—C55—C56	120.6 (2)	C63—C62—H62	120.00
C51—C56—C55	120.5 (2)	C62—C63—H63	120.00
C6—C61—C62	119.88 (16)	C64—C63—H63	120.00
C6—C61—C66	121.54 (19)	C63—C64—H64	120.00
C62—C61—C66	118.39 (17)	C65—C64—H64	120.00
C61—C62—C63	120.57 (18)	C64—C65—H65	120.00
C62—C63—C64	120.4 (2)	C66—C65—H65	120.00
C63—C64—C65	119.8 (2)	C61—C66—H66	120.00
C64—C65—C66	120.4 (2)	C65—C66—H66	120.00
			
C6—N1—C2—C3	35.9 (3)	C2—C21—C22—Cl1	−0.7 (4)
C6—N1—C2—C21	161.74 (19)	C2—C21—C22—C23	−179.8 (3)
C2—N1—C6—C5	1.1 (3)	C26—C21—C22—Cl1	179.7 (2)
C2—N1—C6—C61	−175.12 (18)	C26—C21—C22—C23	0.5 (4)
C5—N4—C3—C2	35.6 (3)	C2—C21—C26—C25	179.9 (2)
C5—N4—C3—C31	164.2 (2)	C22—C21—C26—C25	−0.5 (4)
C3—N4—C5—C6	1.5 (3)	Cl1—C22—C23—C24	−179.0 (3)
C3—N4—C5—C51	−176.3 (2)	C21—C22—C23—C24	0.2 (5)
N1—C2—C3—N4	−54.1 (2)	C22—C23—C24—C25	−1.0 (5)
N1—C2—C3—C31	−179.0 (2)	C23—C24—C25—C26	1.1 (5)
C21—C2—C3—N4	−177.59 (17)	C24—C25—C26—C21	−0.3 (4)
C21—C2—C3—C31	57.5 (3)	C5—C51—C52—C53	−173.11 (19)
N1—C2—C21—C22	130.8 (3)	C56—C51—C52—C53	1.4 (3)
N1—C2—C21—C26	−49.6 (3)	C5—C51—C56—C55	174.06 (18)
C3—C2—C21—C22	−105.1 (3)	C52—C51—C56—C55	−0.6 (3)
C3—C2—C21—C26	74.6 (3)	C51—C52—C53—C54	−1.1 (3)
N4—C5—C6—N1	−22.5 (3)	C52—C53—C54—C55	0.0 (3)
N4—C5—C6—C61	153.6 (2)	C53—C54—C55—C56	0.9 (3)
C51—C5—C6—N1	155.2 (2)	C54—C55—C56—C51	−0.6 (3)
C51—C5—C6—C61	−28.8 (3)	C6—C61—C62—C63	175.09 (18)
N4—C5—C51—C52	142.8 (2)	C66—C61—C62—C63	0.0 (3)
N4—C5—C51—C56	−31.7 (3)	C6—C61—C66—C65	−173.53 (19)
C6—C5—C51—C52	−35.0 (3)	C62—C61—C66—C65	1.5 (3)
C6—C5—C51—C56	150.53 (19)	C61—C62—C63—C64	−1.4 (3)
N1—C6—C61—C62	−38.6 (3)	C62—C63—C64—C65	1.4 (3)
N1—C6—C61—C66	136.3 (2)	C63—C64—C65—C66	0.1 (3)
C5—C6—C61—C62	145.14 (19)	C64—C65—C66—C61	−1.6 (3)
C5—C6—C61—C66	−39.9 (3)		

### Hydrogen-bond geometry (Å, °)

**Table:**

Cg2, Cg3 and Cg4 are the centroids of the C21–C26, C51–C56 and C61–C66 rings, respectively.

**Table:**

*D*—H···*A*	*D*—H	H···*A*	*D*···*A*	*D*—H···*A*
C24—H24···Cg4^i^	0.93	2.80	3.643 (3)	152
C53—H53···Cg2^ii^	0.93	2.99	3.873 (4)	159
C64—H64···Cg3^iii^	0.93	2.88	3.729 (2)	153

Symmetry codes: (i) −*x*+1, −*y*, −*z*+1; (ii) −*x*, −*y*, −*z*; (iii) *x*, −*y*+1/2, *z*+1/2.
